# Viral acute respiratory illnesses in elite athletes: A 12-month controlled follow-up study

**DOI:** 10.1371/journal.pone.0322283

**Published:** 2025-06-02

**Authors:** Raakel Luoto, Vesa Laatikainen-Raussi, Katja E. Mjøsund, Maarit Valtonen, Matti Uhari, Johanna K. Ihalainen, Tytti Vuorinen, Antti Hakanen, Matti Waris, Olli J. Heinonen, Olli Ruuskanen

**Affiliations:** 1 Department of Paediatrics and Adolescent Medicine, Turku University Hospital and University of Turku, Turku, Finland; 2 Finnish Institute of High Performance Sport KIHU, Jyväskylä, Finland; 3 Biology of Physical Activity, Faculty of Sport and Health Sciences, University of Jyväskylä, Jyväskylä, Finland; 4 Paavo Nurmi Centre and Unit for Health and Physical Activity, University of Turku, Turku Finland; 5 PEDEGO Research Unit, University of Oulu and Department of Paediatrics and Adolescents, Oulu University Hospital, Oulu, Finland; 6 Institute of Biomedicine, University of Turku and Department of Clinical Virology, Turku University Hospital, Turku, Finland; United States Environmental Protection Agency, UNITED STATES OF AMERICA

## Abstract

**Background:**

Viral acute respiratory illnesses (ARIs) are the most common acute illnesses in elite athletes. However, the occurrence, aetiology, and clinical manifestations of viral ARIs in athletes remain unclear.

**Methods:**

Twenty-four elite cross-country skiers and 22 elite orienteers were followed for 12 months. Thirty-two normally exercising, healthy young adults were recruited as controls. Occurrences of ARI symptoms were collected weekly with a digital questionnaire. Nasal swabs for respiratory viruses were collected at the onset of symptoms and once monthly when asymptomatic.

**Results:**

A significantly higher incidence density (per person per year) of ARI during the 12-month follow-up period was detected in the skiers compared to the controls (mean (SD) 3.39 (2.13) vs. 2.11 (1.98), respectively, p = 0.037) whereas the differences between the skiers and orienteers (mean (SD) 2.39 (1.07)) and between the orienteers and controls did not reach statistical significance (p = 0.053 and 0.506, respectively). The COVID-19 pandemic prevention measures and lockdown dramatically eliminated the occurrence of ARIs in all study groups. ARI episodes were shorter and milder in the orienteers (not studied in the skiers) compared to the controls (p = 0.001 and p = 0.001). A combination of international flights and participation in a competition was associated with a significant risk of an ARI episode in the skiers (p = 0.048). Rhinoviruses (54.1%) and seasonal coronaviruses (21.6%) were the most common viruses detected in all study groups.

**Conclusion:**

The incidence of ARIs was higher among the skiers compared to the orienteers and the controls. However, ARI episodes were shorter and milder in the orienteers compared to the controls.

## 1. Introduction

Elite athletes are predisposed to several risk factors that may increase their susceptibility to viral acute respiratory illnesses (ARIs) [1,2]. It is well documented that strenuous physical stress may induce a relative immunosuppression [3–5]. However, the clinical meaning of the immunosuppression is not known [2,6,7]. Susceptibility to a viral ARI is a complex combination of epidemiological, immunological, environmental, and behavioral factors [8]. The role of a single factor in resistance to an ARI is difficult to demonstrate. Importantly, respiratory viruses circulate in the community mostly during autumn and wintertime and then the risk of transmission is highest [9–13]. Respiratory viruses spread especially in closed environments, crowded indoor spaces, and closed-contact settings. Heavy breathing, talking, singing, and exercising enhance transmission [14–17]. Shared housing and meals are other risk factors. Elite athletes are often subject to most of these ARI risk factors [2,18]. However, the occurrence of ARIs in athletes is still poorly understood [19,20].

The aims of this prospective controlled 12-month follow-up study were to investigate the occurrence, etiology, risk factors, and clinical manifestations of ARIs in elite endurance sport athletes. Importantly, respiratory viruses were searched for at the onset of ARI symptoms and monthly when asymptomatic.

## 2. Methods

### 2.1. Study design and participants

This prospective observational study was carried out between September 1, 2019, and August 31, 2020. The athletes of the Finnish National Nordic Ski Team (winter sport, n=24) and Finnish National Orienteering Team (summer sport, n=22) for the 2019/2020 competitive season were recruited to this study. All athletes were defined as elite-level athletes and were training on average 450–800 hours annually [21]. As controls, we recruited 37 normally exercising (<6 hours/week) subjects from the students and staff of Turku University Hospital and University of Turku. The recruitment period for this study was from August 1–31, 2019. The team physicians (KM, MV) interviewed the athletes, and the study nurse the controls during the first study visit. The clinical characteristics of the study subjects are presented in Table 1.

**Table 1:** Clinical characteristics of the study population.

	Skiers (n = 24)	Orienteers (n = 22)	Controls (N=32)	p value (95% CI)
Sex (Male: Female)	13 (54%): 11 (46%)	8 (36%): 14 (64%)	10 (31%): 22 (69%)	0.232
Age	25.9 (20.4–35.7)	25.2 (18.7–32.6)^a^	29.5 (20.0–42.5)^a^	0.010 (25.7‒28.4)
*Female*	*26.0 (20.4–35.7)*	*25.6 (18.7–32.6)*	*28.6 (20.0–40.3)*	*0.261 (25.1*‒*28.9)*
*Male*	*25.7 (22.5–32.2)* ^*a*^	*24.7 (22.5–29.4)* ^*b*^	*31.3 (23.3–42.5)* ^*ab*^	*0.006 (25.2*‒*29.1)*
Body mass (kg)	68.0 (51.0–85.0)	61.8 (50.0–80.0)^a^	71.7 (52.0–112.0)^a^	0.031 (65.1‒71.1)
*Female*	*60.1 (51.0–67.0)*	*56.3 (50.0–66.0)* ^*a*^	*64.8 (52.0–93.0)* ^*a*^	*0.021 (59.0*‒*64.3)*
*Male*	*75.*2 *(66.0–85.0)*^*a*^	*68.*8 *(60.0–80.0)*^*b*^	*86.*9 *(52.0–112.0)*^*ab*^	*0.003 (72.7*‒*82.0)*
Height (cm)	175 (161–189)	175 (162–191)	172 (154–192)	0.544 (172‒176)
*Female*	*170 (161–177)*	*169 (162–181)*	*169 (154–180)*	*0.945 167*‒*171)*
*Male*	*180 (171–189)*	*182 (178–191)*	*180 (163–192)*	*0.814 (178*‒*183)*
Asthma diagnosis	10 (42%)	8 (36%)	2 (6%)	0.769
*Female*	*4 (36%)*	*6 (43%)*	*1 (5%)*	*–*
*Male*	*6 (46%)*	*2 (25%)*	*1 (10%)*	*–*
Children at home (yes)	1 (4%)^a^	1 (5%)^b^	11 (34%)^ab^	0.003
*Female*	*0 (0%)*	*1 (5%)*	*7 (22%)*	*–*
*Male*	*1 (4%)*	*1 (4%)*	*4 (13%)*	*–*
Exercise load (h)	Yearly 808 (651–952)^a^	Yearly 475 (256–595)^b^	Weekly <6^ab^	< 0.001 (558‒712)
*Female*	*755 (651–864)* ^*a*^	*445 (256–575)* ^*b*^	*Weekly <6* ^*ab*^	*< 0.001 (479*‒*666)*
*Male*	*869 (800–952)* ^*a*^	*550 (464–595)* ^*b*^	*Weekly <6* ^*ab*^	*< 0.001 (617*‒*866)*

Values are represented as mean (range) or as number (%). Group comparisons were made with ANOVA (except sex, asthma diagnosis, and children at home with Fisher Freeman Halton exact test) and pairwise comparisons with Bonferroni. Superscript a and b show pairwise significant difference.

This study complied with the Declaration of Helsinki as revised in 2000 and all study-related activities were conducted according to Good Clinical Practice. The study protocol was approved by the Ethics Committee of the Hospital District of Southwest Finland (ETMK Dnro: 5/ 1801/ 2019). Written informed consent was obtained from all study subjects.

### 2.2. Data collection

At the onset of ARI, the participants were instructed to fill a structured daily symptom diary and to take a nasal swab sample. Additionally, every Monday morning throughout the 12-month study period a text-message questionnaire was distributed automatically to the study subjects. The digital questionnaire included the occurrence of ARI symptoms during the past seven days. The team physicians (KM and MV) interviewed the skiers and the orienteers personally and confirmed the occurrence of ARI symptoms. Additionally, weekly data on international air travel and participation in competitions was collected. All athletes were requested to maintain their normal training and competition diaries throughout the 12-month study period.

### 2.3. Assessment of illness

ARI was defined as the acute onset of at least one of the following symptoms lasting at least one day: sore throat, rhinorrhea, nasal congestion, cough, and fever (≥37.8˚C) [22]. A 4-point severity scale (0 = absent, 1 = mild, 2 = moderate, and 3 = severe) was used. The total symptom score of fever, sore throat, rhinorrhea, nasal congestion, cough, hoarseness, lethargy, and muscle soreness (max 105 points) for the first five days of illness was calculated for the orienteers and controls [23].

### 2.4. Sample collection

Flocked nasal swabs (553C, Copan, Brescia, Italy) from a depth of 4–5 cm were taken on days 1–3 of the illness. The orienteers and controls took follow-up samples between the days 14–21 of the illness. In addition, nasal swabs were taken at the beginning of every month if the study subject was asymptomatic and had been a minimum of seven days asymptomatic after the last symptomatic infection.

During the prepandemic period, the study subjects contacted the study nurse at the onset of ARI symptoms and a study visit was organized for sample collection during the first three days of illness. During the pandemic period, no study visits were allowed, and the study subjects self-collected the samples. The swabs were mailed to the laboratory and stored at -70°C until analysis [24].

### 2.5. Viral studies

Laboratory testing was carried out for respiratory syncytial type A and B viruses, adenoviruses, influenza A and B viruses, rhinoviruses, enteroviruses, parainfluenza type 1–4 viruses, human coronaviruses 229E, OC43, and NL63, human bocaviruses, and human metapneumoviruses (Allplex Respiratory Panels 1–3, Seegene, Seoul, South Korea). In addition, a more sensitive in-house triplex PCR assay was used for rhinoviruses, enteroviruses, and respiratory syncytial viruses [25]. SARS-CoV-2 viruses were detected using PCR assay [26].

### 2.6. Statistical analyses

An ARI episode was defined as starting in the week when the symptoms occurred and lasting as many weeks as the symptoms continued. At least one asymptomatic week was required before a new episode. Viral detection episodes were defined similarly in weeks in which the virus was detected, and these episodes were classified as symptomatic or asymptomatic.

Data on health status was analyzed as observation weeks. The observation time was defined as all weeks with available and acceptable data. If the observation time was under 50% of the follow-up time, the person was considered as a dropout in the analysis. Subjects with multiple deficiencies in their data and several missing monthly asymptomatic and symptomatic samples, were considered as dropouts.

For each subject an incidence density (ID) was calculated separately for ARI episodes, virus-positive asymptomatic episodes, and viral detection episodes. Due to the exceptional circumstances during the COVID-19 pandemic, we conducted the ID analysis for episodes separately for 12 months, prepandemic, lockdown, and postlockdown periods. All episode IDs were calculated by dividing the number of weeks by the number of total observation times in weeks. For all IDs, the results were multiplied by 52 to achieve a result corresponding to episodes per person per year (ppy). To determine the risk of international air travel and competitions for ARIs, individual IDs were separately calculated for weeks with ARI symptoms without and with air traveling and/or competition during the preceding week. The differences between these individual IDs were analyzed with paired t-test.

Results for symptoms are presented as median (IQR). Other results are presented as mean (SD), mean (range), or number (%). Continuous variables were analyzed with parametric tests and correlations with the Spearman correlation test. Pairwise analysis was performed with t-tests if multiple variance analysis (with Bonferroni correction) showed significant differences between groups.

## 3. Results

**Fig 1:**
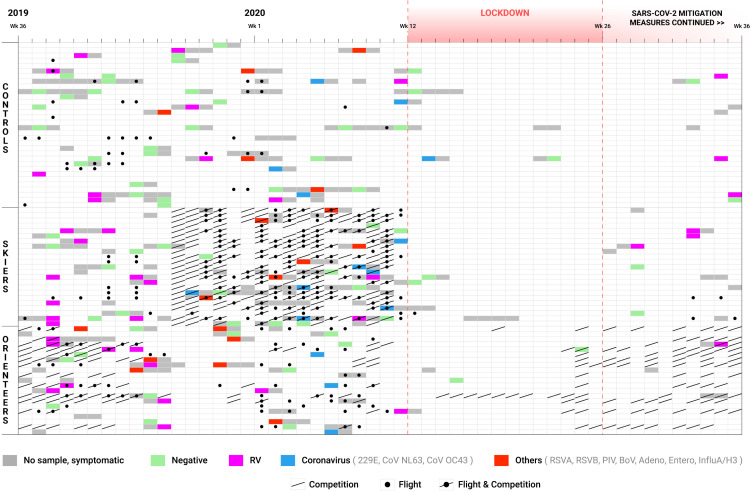
Acute respiratory illnesses (ARIs) during the 12-months study period. Acute respiratory illnesses (ARIs) in the skiers, the orienteers, and the controls during the 12-month study period (between September 1, 2019, and August 31, 2020). The viral samples taken are presented with separate colors depending on the result (grey – no sample, symptomatic; green – negative; purple – RV (rhinoviruses); blue – Coronavirus (human coronaviruses 229E, OC43, and NL63); red – others (RSVA & RSVB - respiratory syncytial type A and B viruses, PIV - parainfluenza type 1–4 viruses, BoV - human bocaviruses, Adeno - adenoviruses, Entero - enteroviruses, InfluA/H3 - influenza A viruses. International flights and competitions are also presented.

The first 29 weeks of the follow-up period (from September 1, 2019, to March 12, 2020, i.e., prepandemic period) were normal, however, the latter part of the follow-up period (from March 13 to August 31, 2020) was influenced by the COVID-19 pandemic; this included the lockdown period (in Finland from March 13 to June 15, 2020) (Fig 1). The skiers´ training season had started at the beginning of May 2019 and the competition season started at the beginning of November 2019. The international competition season was suspended on March 1, 2020. The orienteers started this study during their international competition season, which had started on April 2019. The competition season ended in the end of October 2019 followed by the training season. The next competition season started on April 2020, which was modified by the COVID-19 pandemic.

### 3.1. Return of the study materials

**Fig 2:**
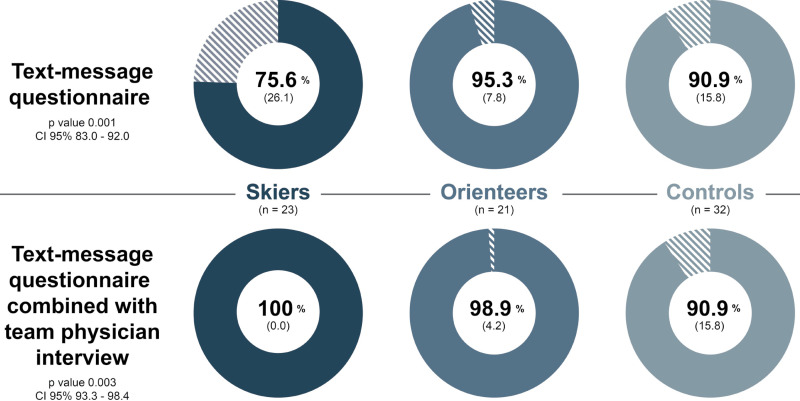
The response rates to weekly symptom surveys in the three study groups. Values are represented as mean (SD), ANOVA-test. Independent T-test showed significant difference for text-message questionnaire between skiers and orienteers (p = 0.002), and skiers and controls (p = 0.018), but not between orienteers and controls (p = 0.178). Independent T-test showed significant difference for text-message questionnaire combined with team physician interview between skiers and controls (p = 0.003), and orienteers and controls (p = 0.009), but not between skiers and orienteers (p = 0.215).

The mean (SD) response rate (self-reported combined with team physician interview) to weekly symptom surveys was 95.9% (11.2) (Fig 2). Altogether five controls, who did not follow the study protocol, were excluded and therefore the final number of controls in the analysis was 32. Additionally, one skier and one orienteer were considered dropouts. The exclusion was done before any analyses. 836 nasal swabs were tested for viruses as follows: 140 samples taken at the beginning of ARI symptoms, 73 follow-up samples, and 623 as asymptomatic samples.

### 3.2. Occurrence of the ARIs

There were 3913 potential person-weeks of observation and in 353 (9.0%) of the weeks the study subjects reported symptoms of an ARI (Fig 1). The 353 weeks with symptoms included 194 distinct illness episodes. Study subjects experienced a mean of 2.6 (range 0‒9) ARI episodes ppy. A significant difference was detected among the study groups in the incidence of ARI episodes during the 12-month follow-up, with the mean (SD) being 3.39 (2.13) in the skiers, 2.39 (1.07) in the orienteers, and 2.11 (1.98) in the control group (p = 0.037) (Table 2). The incidence was significantly higher in the skiers compared to the controls (p = 0.025) but not between the skiers and the orienteers nor between the orienteers and the controls. During the prepandemic, lockdown, and postlockdown periods, significant differences were found only during the prepandemic period (Table 2). Again, the skiers had a significantly higher incidence of ARI compared to the controls (p = 0.016). The differences between the skiers and the orienteers and the orienteers and the controls were not significant. Neither sex nor age explained the incidence of ARIs, asymptomatic infections or viral detection episodes in the whole study population. In the subgroup analysis, only significant difference between sexes was found in the control group, where women were detected to have more asymptomatic infections than men (p = 0.017). All the athletes reported at least one ARI episode while seven (22%) of the controls did not report ARI symptoms during the 12 months (Table 3). The COVID-19 pandemic prevention procedures had a similar effect on all study groups, that is, they nearly eliminated the occurrence of ARIs (Table 2, Fig 1).

**Table 2:** Incidence density (ppy) of acute respiratory illness (ARI) episodes, virus positive asymptomatic episodes, and viral detection episodes a) during the 12-month follow-up period in the skiers, the orienteers, and the controls and b) separately during the prepandemic, lockdown, and postlockdown periods in the skiers, the orienteers, and the controls.

a.	Skiers	Orienteers	Controls	p value (95% CI)
ARI episodes	3.39 (2.13)^*^	2.39 (1.07)	2.11 (1.98)^*^	0.037 (2.14‒3.00)
Virus positive asymptomatic episodes	0.91 (1.04)^*^	0.29 (0.47)^*^	0.54 (0.64)	0.023 (0.41‒0.76)
Viral detection episodes	2.13 (1.49)^*^	1.34 (0.92)^*^	1.34 (1.27)^*^	0.046 (1.28‒1.87)
**b.**	**Skiers**	**Orienteers**	**Controls**	**p value**
ARI episodes				
*Prepandemic*	5.33 (3.28)^*^	3.91 (1.66)	3.27 (2.84)^*^	0.025 (3.44‒4.72)
*Lockdown*	0.32 (1.07)	0.53 (1.33)	0.36 (1.47)	0.847 (0.09‒0.69)
*Postlockdown*	2.26 (2.64)	0.74 (1.86)	1.34 (2.31)	0.094 (0.92‒2.00)
*p value*	*<0.001*	*<0.001*	*<0.001*	
Virus positive asymptomatic episodes				
*Prepandemic*	1.45 (1.85)^*^	0.45 (0.82)^*^	0.85 (1.07)	0.039 (0.60‒1.22)
*Lockdown*	0.32 (1.07)	0.18 (0.81)	0.36 (1.12)	0.825 (0.06‒0.52)
*Postlockdown*	0.23 (1.08)	0.00 (0.00)	0.00 (0.00)	0.327 (-0.07-0.21)
*p value*	*0.009*	*0.118*	*0.005*	
Viral detection episodes				
*Prepandemic*	3.47 (2.70)^*^	2.31 (1.66)	2.02 (1.95)^*^	0.042 (2.04‒3.04)
*Lockdown*	0.32 (1.07)	0.18 (0.81)	0.48 (1.27)	0.642 (0.10‒0.59)
*Postlockdown*	0.90 (2.02)	0.25 (1.13)	0.67 (1.77)	0.438 (0.23‒1.02)
*p value*	*<0.001*	*<0.001*	*<0.001*	

The values are presented as mean (SD), ANOVA test. Pairwise analysis between groups was performed with T-tests. ^*^p = <0.05. Tests between different time periods were performed with repeated measures ANOVA with Bonferoni correction in pairwise comparison. ppy – per person per year.

**Table 3:** The number (0/ ≥1 ppy) of acute respiratory illness (ARI) episodes, virus positive asymptomatic episodes, and viral detection episodes during the 12-month follow-up period in the skiers, the orienteers, and the controls.

	Skiers (n = 23)	Orienteers (n = 21)	Controls (n = 32)	p value
ARI episodes				
0	0 (0%)	0 (0%)	7 (22%)	
≥1	23 (100%)	21 (100%)	25 (78%)	0.006
Virus positive asymptomatic episodes				
0	10 (43%)	15 (71%)	17 (53%)	
≥1	13 (57%)	6 (29%)	15 (47%)	0.178
Viral detection episodes				
0	1 (4%)	5 (24%)	8 (25%)	
≥1	22 (96%)	16 (76%)	24 (75%)	0.093

The values are presented as number (%), Fisher-Freeman-Halton Exact Test. ppy – per person per year.

### 3.3. Etiology of the ARIs

A nasal swab sample was taken in 140 (72.2%) of the 194 ARI episodes. The viral etiology was identified in 74 (52.9%) samples without significant differences among the study groups. Rhinoviruses (n = 40, 54.1%) and seasonal coronaviruses (n = 16, 21.6%) were the most common viruses detected in all study groups (Table 4). The numbers of subjects with a high viral load (Ct-values <27) did not differ among the study groups (Table 4).

**Table 4:** Viral findings for the skiers, the orienteers, and the controls separately when having symptoms of an acute respiratory illness (ARI) and during asymptomatic phase.

Virus	Total n (%)	Skiers	Orienteers	Controls	p value
	ARI/ asymptomatic	(n = 23)	(n = 21)	(n = 32)	
Adenovirus	1 (1.4)/ 0 (0)	1/ 0	0/ 0	0/ 0	NS
Coronavirus type 229E	3 (4.1)/ 0 (0)	1/ 0	1/ 0	1/ 0	NS
Coronavirus type NL63	6 (8.1)/ 4 (7.8)	5/ 2	0/ 1	1/ 1	NS
Coronavirus type OC43	7 (9.5)/ 4 (7.8)	2/ 4	2/ 0	3/ 0	NS
Influenza A virus	5 (6.8)/ 2 (3.9)	2/ 2	2/ 0	1/ 0	NS
Influenza B virus	0 (0)/ 0 (0)	0/ 0	0/ 0	0/ 0	–
Human metapneumovirus	0 (0)/ 0 (0)	0/ 0	0/ 0	0/ 0	–
Parainfluenza virus type 1	5 (6.8)/ 3 (5.9)	1/ 1	3/ 1	1/ 1	NS
Parainfluenza virus type 2	0 (0)/ 0 (0)	0/ 0	0/ 0	0/ 0	–
Parainfluenza virus type 3	0 (0)/ 0 (0)	0/ 0	0/ 0	0/ 0	–
Parainfluenza virus type 4	2 (2.7)/ 0 (0)	0/ 0	0/ 0	2/ 0	NS
RSV group A	2 (2.7)/ 3 (5.9)	0/ 1	1/ 0	1/ 2	NS
RSV group B	1 (1.4)/ 0 (0)	0/ 0	1/ 0	0/ 0	NS
Rhinovirus	40 (54.1)/ 33 (64.7)	15/14	11/ 4	14/ 15	NS
Human enterovirus	2 (2.7)/ 0 (0)	1/ 0	1/ 0	0/ 0	NS
Human bocavirus	0 (0)/ 2 (3.9)	0/ 0	0/ 1	0/ 1	NS
SARS-CoV-2	0 (0)/ 0 (0)	0/ 0	0/ 0	0/ 0	
*Number (%) of ARI samples*	*with Ct-value <27*	*12 (43%)*	*10 (45%)*	*6 (25%)*	*NS*

Two viruses were detected in the same sample as follows: Rhinovirus 4 times, Influenza A virus 2 times, and Human bocavirus 1 time. NS = Not significant.

From the follow-up nasal samples (n = 73) taken by orienteers (n = 24) and controls (n = 49) between days 14–21 of the ARI, a total of 11 viruses were detected. Rhinovirus was the most detected virus (10/11, 91%). Of these 11 samples, three samples from the orienteers and two samples from the controls were positive for rhinovirus after the first sample taken at the onset of the symptoms was negative.

### 3.4. Clinical manifestation of the ARIs in the orienteers and the controls

Symptom diaries were available for 47 (71%) ARI episodes of the controls and for 32 (64%) of the orienteers. The median duration of the symptoms was 4 (IQR, 3–8) days and 11 (IQR, 7–13) days in the orienteers and the controls, respectively (p = 0.001). The symptoms were mostly mild, and the total median severity score was 14.5 (IQR, 7–25) for the orienteers and 24.0 (IQR, 15–35) for the controls (p=0.001). Five febrile illness episodes were reported (one in the orienteers and four in the controls).

### 3.5. Viral detection episodes

There were 125 viral detection episodes (mean 1.6 (range 0–5) ppy). The skiers had significantly more viral detection episodes (ppy) compared to the orienteers and the controls (p = 0.038 and 0.038, respectively) (Table 2). Of 125 viral detection episodes, 74 (59.2%) were associated with ARI symptoms. There was no significant difference between study groups in the incidence of symptomatic viral detection episodes.

A virus was detected in 51 (8.1%) of the 623 asymptomatic phase samples. Rhinovirus was the most detected virus (n = 33, 64.7%), followed by seasonal coronaviruses (n = 8, 15.7%) (Table 4). The skiers had significantly more virus-positive asymptomatic episodes compared to the orienteers (p = 0.014) whereas the differences between the skiers and the controls or between the orienteers and the controls were not significant (Table 2). In the further analysis, the only significant difference in the incidence of virus-positive asymptomatic episodes was between the skiers and the orienteers during the prepandemic period (p = 0.024) (Table 2).

### 3.6. Risk factors for ARI episodes

International flights and/or competitions were associated with a significant risk for an ARI episode in the skiers (11.9% vs 6.3%, p = 0.048), but not in the orienteers and the controls.

## 4. Discussion

We found that the incidence of ARIs was significantly higher among the skiers compared to the orienteers and the controls during the 12-month follow-up period. In addition, the occurrences of virus-positive symptomatic and asymptomatic episodes were higher in the skiers. COVID-19 prevention procedures nearly eliminated the occurrence of ARIs in all study groups. Rhinoviruses and seasonal coronaviruses were the most common causative agents. International flights and/or participation in competitions were a significant risk factor for contraction of ARI in the skiers.

ARIs are the most frequently reported non-injury-related illnesses in athletes [20]. The real burden of ARIs in athletes has not been identified due to the lack of prospective, controlled cohort studies with virus diagnostics [7,19,20]. Recently, a systematic review of 124 studies by the International Olympic Committee (IOC) reported the annual ARI incidence to be 1.7 in athletes [20]. This is lower or comparable with that of the normal population [27,28]. In one study the young adult population experienced 2–6 ARIs annually [27]. In our study, the incidence of ARI was highest in the skiers. During the prepandemic period, (i.e., normal life), the annual incidence of ARIs was 5.3 for the skiers, 3.9 for the orienteers, and 3.3 for the controls. Prepandemic analysis is, however, probably an overestimation due to high viral activity in the community during that time (the autumn and wintertime) (S1 Fig) [10,11].

COVID-19 mitigation measures induced a remarkable reduction in the occurrence of non-COVID respiratory viruses world-wide. Discontinuation of the interventions led to a concurrent resurgence of the infections [29–31]. Our findings on athletes agree with those observations. The mitigation protocols efficiently reduced the occurrence of ARI episodes as well as virus-positive asymptomatic episodes in all study groups. During the 2018 Winter Olympics, 45% of 44 athletes in Team Finland caught a common cold, while during the COVID-19 mitigation strategies during the 2022 Winter Olympics, the corresponding percentage in 47 athletes was 6% [32,33].

We detected 11 different respiratory viruses in athletes, but rhinoviruses and seasonal coronaviruses were the most commonly detected viruses comprising 78% of the virus-positive findings. This observation agrees with those in the general population [9,11,27]. A viral etiology was detected in only 53% of the 140 samples taken during the symptomatic episodes. This is a lower proportion than in the etiological studies in the athletes and in the general population [9,23,27,32]. The reason for the low etiologic detection rate in our study population may be due to the non-optimal timing of the sample, self-sampling, and specimen quality [24].

Interestingly, the athletes experienced mild ARIs in terms of both the duration and the intensity of the symptoms which is in agreement with results of the systematic review and meta-analysis of the IOC consensus [8,34]. The 4-day duration agrees with the observations of earlier studies in athletes [23,35]. In contrast, the mean duration of the common cold for 199 young adults was 10 days [36]. In a review of 11518 athletes with COVID-19, the clinical presentation was mild or asymptomatic in 94% of cases [37]. In contrast to immunosuppression, regular exercise activates body´s defense systems and may partly explain the mild clinical manifestation of ARIs in athletes [5,6]. Importantly, no bacterial coinfections were diagnosed in this study.

Asymptomatic respiratory viral infections are more common than earlier recognized. The high prevalence of asymptomatic SARS-CoV-2 positivity and transmission of the virus from asymptomatic individuals have been shown in several different studies [38]. In one family study, 44% of the 783 detected viral episodes were asymptomatic [27]. In our previous study among elite skiers, 20% of the viral infections were asymptomatic [23]. The corresponding number in this study was 41%. The most common viruses were rhinoviruses and seasonal coronaviruses as in the general population [9,27]. The clinical impact of asymptomatic viral infections in athletes is not known.

International flights and/or competitions were associated with a significantly increased risk for an ARI in the skiers but not in the orienteers. Our results agree with those of Svendsen et al., who showed in 37 elite cross-country skiers that competition and air travel increased the risk of ARI three- and fourfold, respectively [18]. In our previous two studies, 8% of Team Finland members traveling to major winter sports events contracted ARI most probably during air travel [23,32]. We analysed air travel and competitions as a combination since due to the 2–6 days incubation period of respiratory viruses, it is difficult to prove the moment of contraction and thus also the source of transmission [39].

It is tempting to link competition with heavy physical and mental stress and thus to enhanced susceptibility to ARIs. However, both traveling to and participating in competitions are intimately connected to human crowding [40]. The time spent in a crowded indoor space is the key determinant in the transmission of respiratory viral infections, which occur mainly through infectious respiratory particles [14,15,17,41]. Air travel and even short bus rides are under-recognized risk factors for contraction of ARI [16,40,42]. Furthermore, during winter months the risk for viral ARI is higher due to the marked viral pressure in the community, thus explaining the putatively significant risk of winter sport athletes for ARIs [10‒13].

Our study has certain limitations. The number of participants is low due to the limited number of athletes in the national teams. The study population included only endurance discipline athletes and for example team-sport and high-intensity exercise athletes were not studied. The samples were self-collected. The 12-month study period was unpredictably influenced by COVID-19 restrictions and only the first six months of the follow-up represented the normal seasonality of respiratory viruses. Our observations may be biased by the different number of young children in the study groups; 34% of the controls had children at home, compared to 4% in the skiers and 5% in the orienteers. Having young children at home is the most important risk factor for contraction of ARI and this may underestimate the difference in ARI rates between the athletes and the controls [27,41]. The athletes suffered more from asthma which may have influenced the occurrence of respiratory viral infections. In this study, we analyzed only two well-defined risk factors and the role of other possible risk factors like heavy physical exercise and psychological stress remain unclear.

In conclusion, our findings suggest that the skiers suffer from more viral ARIs than non-athletic subjects. The clinical manifestations of viral ARIs in athletes are most often mild. The skiers travel frequently and are exposed to crowding during the wintertime when the community pressure of viruses is high. COVID-19 mitigation measures effectively reduced the occurrence of ARIs also in these athletes. Therefore, intensified infection control measures during travelling and competitions are recommended.

## Supporting information

S1 Fig Seasonality of respiratory viruses. Weekly numbers of viral detections during the 12-month study period (between September 1, 2019, and August 31, 2020) in Turku University Hospital Laboratories, Department of Clinical Microbiology. AdV – adenoviruses, EV – enteroviruses, HBoV - human bocaviruses, HCoV – human coronaviruses 229E, OC43, and NL63, InfA & InfB - influenza A and B viruses, MPV - human metapneumoviruses, PIV 1–4 - parainfluenza type 1–4 viruses, RSV - respiratory syncytial type A and B viruses, RV – rhinoviruses, SARS-CoV-2 – severe acute respiratory syndrome coronavirus.
(TIF)

S1 Table Summary of the key findings of statistical comparison between the study groups. (DOCX)

S1 File STROBE statement – checklist of items that should be included in reports of cohort studies. (DOCX)
