# Supplementary figures and images for: Viral acute respiratory illnesses in elite athletes: A 12-month controlled follow-up study
Source: PLoS One. 2025 Jun 2;20(6):e0322283. doi: 10.1371/journal.pone.0322283 (PMC12129143; doi:10.1371/journal.pone.0322283)

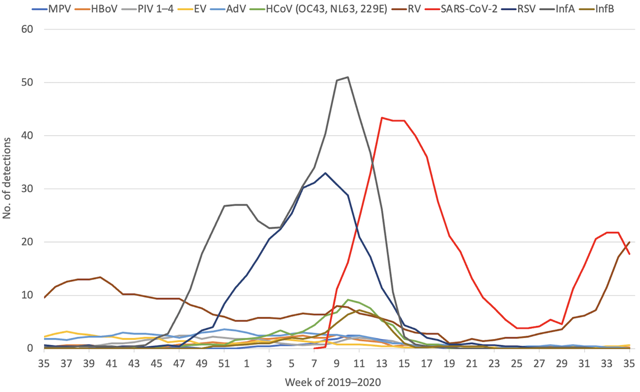

Supplement: S1 Fig — Weekly numbers of viral detections during the 12-month study period (between September 1, 2019, and August 31, 2020) in Turku University Hospital Laboratories, Department of Clinical Microbiology. AdV – adenoviruses, EV – enteroviruses, HBoV - human bocaviruses, HCoV – human coronaviruses 229E, OC43, and NL63, InfA & InfB - influenza A and B viruses, MPV - human metapneumoviruses, PIV 1–4 - parainfluenza type 1–4 viruses, RSV - respiratory syncytial type A and B viruses, RV – rhinoviruses, SARS-CoV-2 – severe acute respiratory syndrome coronavirus. (TIF) [file pone.0322283.s001.tif]
