# Supplementary material for: Viral acute respiratory illnesses in elite athletes: A 12-month controlled follow-up study
Source: PLoS One. 2025 Jun 2;20(6):e0322283. doi: 10.1371/journal.pone.0322283 (PMC12129143; doi:10.1371/journal.pone.0322283)
Supplement: S1 Table — (DOCX) [file pone.0322283.s002.docx]

**S1 Table.** **Summary of the key findings of statistical comparison between the study groups.**

|  | **Skiers (n = 23)** | **Orienteers (n = 21)** | **Controls (n = 32)** | **p value (95% CI)** |
| --- | --- | --- | --- | --- |
| Incidence density (ppy) of ARI episodes (mean (SD)) | 3.39 (2.13)^*^ | 2.39 (1.07) | 2.11 (1.98)^*^ | 0.037 (2.14 – 3.00) |
| Duration of ARI episodes (mean (IQR)) | - | 4 (3–8) | 11 (7–13) | 0.001 |
| Severity of ARI episodes (mean (IQR)) | - | 14.5 (7–25) | 24.0 (15–35) | 0.001 |
| Combination of flight(s) and competition(s) increased the risk of ARI | Yes | No | - |  |

The ppy values are presented as mean (SD), ANOVA test with Bonferroni correction. Pairwise analysis between groups was performed with T-tests. ^*^p = <0.05. Other values are presented as median (IQR), Mann-Whitney U test. ppy – per person per year, ARI – acute respiratory illness.
